# Upregulated TUBG1 expression is correlated with poor prognosis in hepatocellular carcinoma

**DOI:** 10.7717/peerj.14415

**Published:** 2022-12-05

**Authors:** Kainan Zhang, Mengsi Yu, Hui Liu, Zhao Hui, Ning Yang, Xiaojuan Bi, Li Sun, RenYong Lin, Guodong Lü

**Affiliations:** 1 Xinjiang Medical University, Urumqi, Xinjiang, China; 2 State Key Laboratory of Pathogenesis, Prevention, and Treatment of Central Asian High Incidence Diseases, Clinical Medical Research Institute, The First Affiliated Hospital of Xinjiang Medical University, Urumqi, Xinjiang, China; 3 Department of Clinical Laboratory, The First Affiliated Hospital of Xinjiang Medical University, Urumqi, Xinjiang, China; 4 College of Pharmacy, Xinjiang Medical University, Urumqi, Xinjiang, China

**Keywords:** Hepatocellular carcinoma, Ubulin gamma 1, Diagnostic biomarker, Overall survival, Carcinogenesis

## Abstract

**Background:**

Hepatocellular carcinoma (HCC) development is a complex pathological process. Tubulin gamma 1 (TUBG1) plays an oncogenic role in several human cancers; however, its functional role in HCC tumorigenesis remains unknown.

**Methods:**

Herein we first evaluated the gene expression levels of TUBG1 in HCC using data from The Cancer Genome Atlas and Gene Expression Profiling Interactive Analysis databases. We then elucidated the association between TUBG1 gene expression levels and survival rates of patients with HCC. Cell cycle, proliferation, transwell migration, and matrigel invasion assays were used to study the effects of TUBG1 on the malignant phenotypes of HCC cells.

**Results:**

Based on the data obtained from the aforementioned databases and our *in vitro* experiments, TUBG1 was found to be overexpressed in HCC and patients with high TUBG1 expression levels showed a remarkably poor overall survival rate. In addition, the expression of TUBG1 significantly promoted the malignant phenotypes of HCC cells *in vitro*. Gene ontology term enrichment analysis revealed that co-regulated genes were enriched in biological processes mainly involved in chromosome segregation, chromosomal region, and chromatin binding; moreover, Kyoto Encyclopedia of Genes and Genome pathway analysis showed that they were mainly involved in cell cycle, oocyte meiosis, platinum drug resistance, and the p53 signaling pathway.

**Conclusions:**

We report that TUBG1 is an important oncogene in HCC. It promotes HCC progression and may serve as a potential prognostic biomarker for HCC. Future studies are warranted to unveil molecular biological mechanisms underlying TUBG1 carcinogenesis.

## Introduction

Hepatocellular carcinoma (HCC) is the second leading cause of cancer-related deaths worldwide, and it has consequently become a major public health challenge (Sia et al., 2017). Although the prevalence of hepatitis B has been controlled with the popularization of HBV vaccine, the number of HCC patients is still increasing year by year (Liu et al., 2020). The reason probably related to HCV infection and non-alcoholic fatty liver disease (Baffy, Brunt & Caldwell, 2012). HCC is often associated with poor prognosis, and surgery is the only effective treatment method at present, although some drug treatment such as Tyrosine Kinase Inhibitors and immune check point inhibitor related drugs have made great progress, the fatality rate of HCC remains high (Hartke, Johnson & Ghabril, 2017). No sensitive and specific biomarkers currently exist for HCC diagnosis and prognosis assessment (West, Black & Mehta, 2019). Serum alpha-fetoprotein (AFP) is the most widely used serological indicator. However, about 30% to 40% of patients diagnosed with liver cancer have no significant increase in AFP, and the sensitivity of AFP in early-stage liver cancer is only 30% to 40%, while about 20% to 50% of patients with chronic hepatitis and cirrhosis have elevated AFP. Therefore, AFP has the problem of low sensitivity and specificity in the screening of liver cancer, and new serological indicators are needed to supplement the deficiency of AFP (Ueno et al., 2022).

Tubulin gamma 1 (TUBG1) encodes a member of the tubulin superfamily. The encoded protein mediates microtubule nucleation and is required for microtubule formation and cell cycle progression (Wise, Krahe & Oakley, 2000). A few recent studies have reported that its main function is related to neurodevelopmental presentations (Ivanova et al., 2019; Yuen et al., 2019). Maounis et al. (2012) found that γ-tubulin is highly expressed in non-small cell lung cancer. Further, Watanabe et al. (2018) reported the use of γ-tubulin to predict BRCA status, concluding that γ-tubulin immunofluorescence, a functional assessment of BRCA, can be used as a new prospective test of BRCA status. In urothelial carcinoma, γ-tubulin is evidently related to the degree of tumor differentiation (Murata et al., 2020). However, there is no evidence that TUBG1/ γ-tubulin is associated with the prognosis of HCC. Zhang et al. (2019) found an association between Hau3, a prognostic indicator of HCC, and TUBG1 expression. Xing, Yan & Zhou (2018) identified TUBG1 was the one of the upregulated markers in HCC samples. Nevertheless, little remains known about the expression level and clinical significance of TUBG1 in HCC.

Herein we aimed to explore the role of TUBG1 in HCC and evaluate its value as a prognostic marker for HCC.

## Materials & Methods

### Data collection

The Cancer Genome Atlas (TCGA; the reported results are in whole or part based upon the data generated by TCGA Research Network:  https://www.cancer.gov/about-nci/organization/ccg/research/structural-genomics/tcga) was used for the analysis of gene expression profiles of TUBG1. The Gene Expression Profiling Interactive Analysis (GEPIA, http://gepia.cancer-pku.cn/) database was applied to analyze the expression of TUBG1 in the HCC cohort (*n* = 369) and healthy controls (*n* = 160) (Tang et al., 2017). After screening the tumor types to be assessed in our study, TUBG1 expression at different stages and influence on the total survival time were further analyzed. The Human Protein Atlas (http://www.proteinatlas.org) database was used to detect TUBG1 expression in liver tissues (Uhlen et al., 2017).

### Cell culture and antibodies

Human HCC cells HepG2, HUH7, HCC-LM3 and control cells L02 were obtained from the Type Culture Collection Cell Bank. Methods for cell culture have been outlined in prior research (Yu et al., 2021; Zhang et al., 2021). Rabbit polyclonal anti-TUBG1 antibody was purchased from Santa Cruz Biotechnology (Santa Cruz, CA, USA).

### Vector construction, transfection and stable cell line construction

Experimental methods were collected as previously described in AUTHOR_ZHANG (12 August 2021). Specifically, vector construction, transfection and stable cell line construction. Genscript, based in Nanjing, China, produced TUBG1 full-length sequences. Next, the TUBG1 sequences were cloned into the virus (8974bp, Hanbio, shanghai, China) to generate the TUBG1 overexpression vector. RiboBio (Guangzhou, China), produced both the Smart Silencer(ssiTUBG1) and the negative control (NCTUBG1). Sequence: GGTCCAGCCTTACAATTCA, GACGCAGAATGCAGACTGT, GAACCTGTCGCCAGTATGA, GGGACCCTCATCTGCCTTAC, CGCATCTCTTTCTCATATAC, ACTTCTCCTCTTATGAGACT.

### qRT-PCR

Total RNA was extracted using TRIzol (Invitrogen), and RNA purity and concentration were determined using a spectrophotometer (NanoDrop ND-2000, Thermo Fisher Scientific, Waltham, MA, USA). cDNA was synthesized using a commercial kit (MaiGene, Binhai, Tianjin), according to manufacturer instructions. SYBR green RT-PCR was performed to measure mRNA levels, which were then calculated using the 2^−ΔΔCt^ method. The following primers were used: TUBG1, 5′-GAATGCAGACTGTGTGGTGG-3′ (forward) and 5′-GTAGTGAGAGGGGTGTAGCC-3′ (reverse) and GAPDH, 5′-TCAAGAAGGTGGTGAAGCAGG-3′ (forward) and 5′-TCAAAGGTGGAGGAGTGGGT-3′ (reverse).

### Cell proliferation, migration, invasion, apoptosis and cell cycle analysis

The EdU (5-Ethynyl-2′-deoxyuridine) was used to measure cell proliferation. The trans well assay was used to evaluate cell migration. Cell invasion was assessed using the BioCoat Matrigel Invasion Chamber (BD Biosciences) (He et al., 2020). Cell numbers for cell migration and invasion were counted in three random fields (He et al., 2020). Cells were stained with Annexin V and propidium iodide using the Annexin V–FITC Apoptosis Detection kit (Invitrogen), and the percentage of apoptotic cells was examined with flow cytometry (Beckman, USA) (He et al., 2020). For detection of the cell cycle, cells were stained with PI after 48 h of transfection and were examined by FACS (He et al., 2020). All experiments were repeated three times, the specific experimental method can refer to the previous article of the researcher (He et al., 2020; Yu et al., 2015a; Yu et al., 2015b).

### Western blotting

The cells were lysed in a lysis solution containing 1% Triton X-100. With the help of a BCA protein assay kit, we calculated the total protein concentration of the cell lysate (Pierce, Rockford, IL, USA). The samples’ respective protein mixtures were subjected to 10% SDS-PAGE before being transferred to a polyvinylidene fluoride membrane for analysis. Immunoblot analysis using mouse anti-GAPDH and rabbit anti-TUBG1 antibodies was done after the membranes were treated with Tris-buffered saline (TBS) containing 5% non-fat milk powder at 37 °C for 2 h. The membranes were then incubated with an HRP-conjugated polyclonal secondary antibody for 1 h at 37 degrees Celsius after being washed three times with TBS/T buffer. The enhanced plus chemiluminescence assay (Pierce) was used to create the membranes following the manufacturer’s guidelines. Image-Pro Plus 6.0 was then used to perform the statistical analysis of the photographs.

### Functional enrichment analysis

The genes co-regulated by TUBG1 were sorted out (correlation coefficient >0.6 and *P* < 0.05). Gene Ontology (GO) and Kyoto Encyclopedia of Genes and Genome (KEGG) functional enrichment analyses were performed using cluster Profiler (Yu et al., 2012).

### Statistical analysis

Statistical analyses were performed using SPSS 22.0 (IBM SPSS, Chicago, IL, USA) and R 3.6.1 (R Foundation for statistical computing, Vienna, Austria). The *χ*^2^ test was used to detect the correlation between TUBG1 expression and clinical characteristics. Overall survival (OS) was analyzed using Kaplan–Meier survival curves with 95% confidence intervals (CIs), and differences between subgroups were compared using the log-rank test. *P* < 0.05 indicated statistical significance.

## Results

### TUBG1 expression was significantly upregulated in HCC

**Figure 1:**
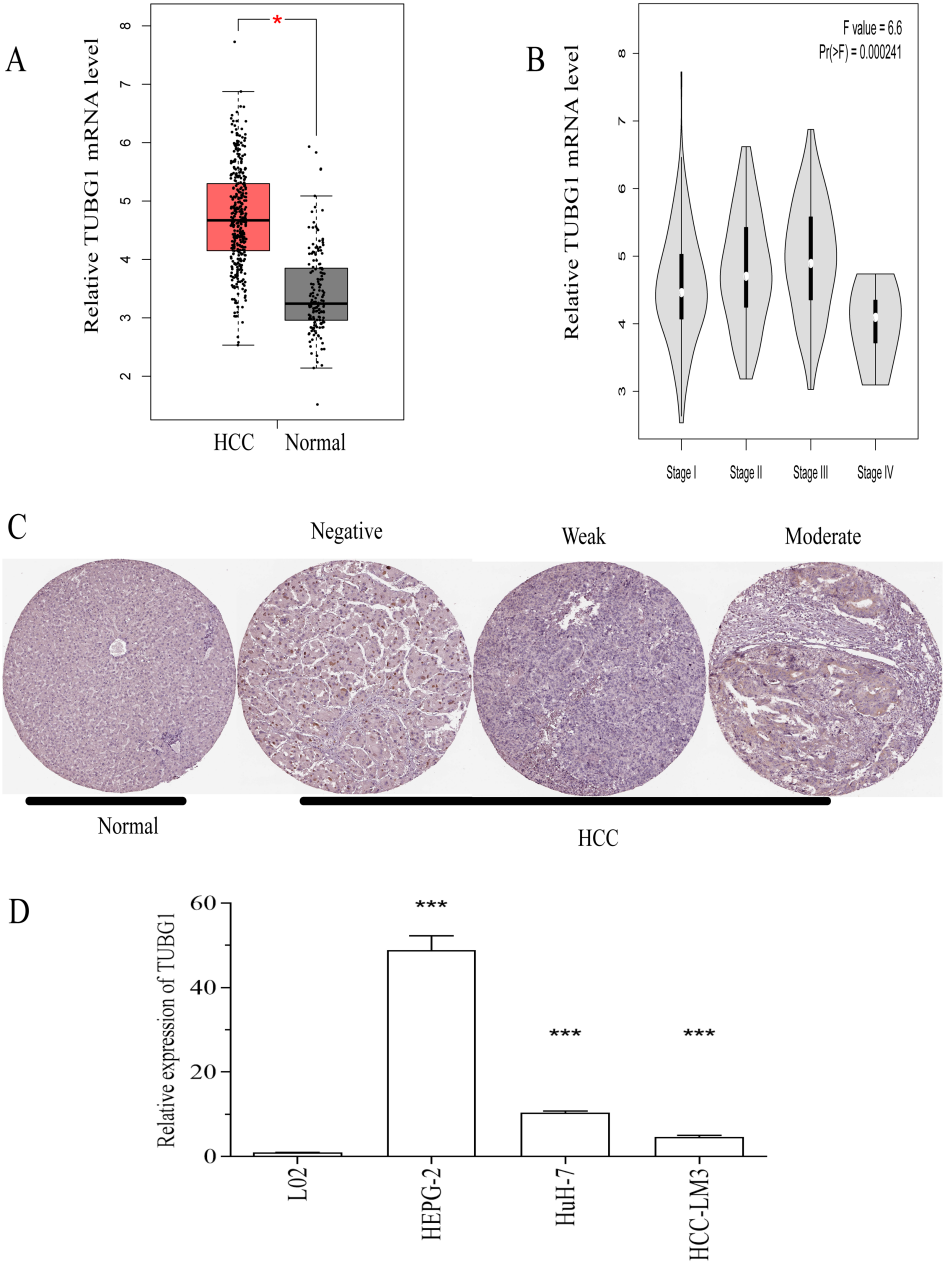
TUBG1 was upregulated in HCC tissues and cell lines. (A) In comparison with adjacent (control) tissues, TUBG1 expression was upregulated in HCC tissues. (B) Expression of TUBG1 was closely associated with the clinicopathological characteristics of HCC. (C) Immunohistochemical staining of TUBG1 (control and HCC tissues). The image is cited from the Human Protein Atlas database, which meets the requirements of the database. Original file: https://www.proteinatlas.org/ENSG00000131462-TUBG1/pathology/liver+cancer. (D) The expression of TUBG1 in L02, HepG2, HUH7 and HCC-LM3 cell lines. TUBG1, tubulin gamma 1; HCC, hepatocellular carcinoma.

To explore the potential role of TUBG1 in HCC, we used the GEPIA database to analyze the expression of TUBG1 and found that the mRNA expression level of TUBG1 was significantly higher in HCC (369 *vs.* 160 in controls, *P* < 0.05; Fig. 1A). Further, TUBG1 expression was different in unequal clinical stages, and the expression levels increased with tumor progression (*P* < 0.001; Fig. 1B, stage IV patients were not included because their sample size was insufficient). The protein expression levels of TUBG1 in HCC and control tissues were assessed using immunohistochemical staining data from The Human Protein Atlas (Uhlén et al., 2015). The representative images indicated that TUBG1 protein expression levels were strongly upregulated in HCC tissues as compared with those in control tissues (Fig. 1C). Testing in different cell lines was performed to elucidate the differential expression of TUBG1 in different HCC cell lines and normal hepatocyte cell lines. The result showed that TUBG1 is down-regulated in L02 cells. In HCC cell lines, the expression of TUBG1 in the HepG2 cell line is significantly higher than that of HUH7 and HCC-LM3 (Fig. 1D). We also facilitate the online database search for the expression of TUBG1 in other cell lines (Fig. S1).

### High TUBG1 expression was correlated with poor clinicopathological characteristics

To further explore the role of TUBG1 in HCC, we performed a preliminary analysis to determine whether the expression of TUBG1 in HCC tissues was associated with clinicopathological characteristics. According to the median value of TUBG1 expression, patients with HCC were divided into high and low TUBG1 expression groups. *χ*^2^ test results revealed the presence of a relationship between TUBG1 expression and clinical characteristics. In patients with HCC, high TUBG1 expression levels were significantly related to clinical stage (*P* < 0.001), tumor status (stage T, *P* = 0.004), race (*P* < 0.001), and vital status (*P* = 0.006; Table 1).

### TUBG1 as a predictor of poor prognosis in patients with HCC

**Figure 2:**
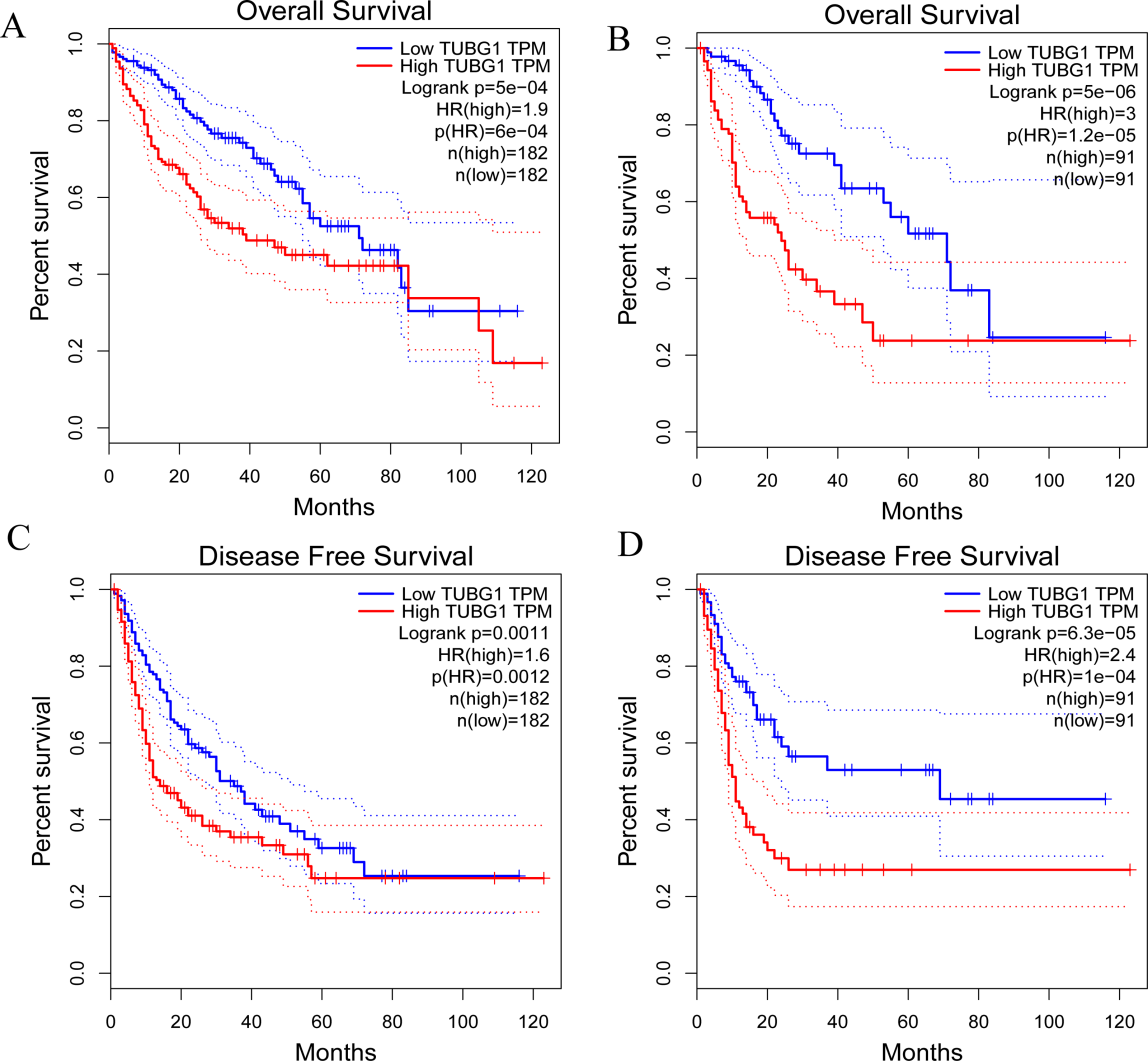
Prognostic significance of TUBG1 in patients with HCC. (A) Kaplan–Meier curves showing the OS of patients with HCC with high and low TUBG1 expression levels, with the median value as the cutoff and (B) with the quartile value as the cutoff. (C) Kaplan–Meier curves showing the DFS of patients with HCC with high and low TUBG1 expression levels, with the median value as the cutoff and (D) with the quartile value as the cutoff.TUBG1, tubulin gamma 1; HCC, hepatocellular carcinoma; OS, overall survival; DFS, disease-free survival.

The prognostic effects of TUBG1 on OS and disease-free survival (DFS) were evaluated *via* Kaplan–Meier survival analysis and the log-rank test. Patients with HCC with upregulated expression levels of TUBG1 showed shorter OS (Figs. 2A, 2B) and DFS (Figs. 2C, 2D) than those with downregulated expression levels of TUBG1 (*P* < 0.001).

### TUBG1 promoted cell proliferation, migration, and invasion and inhibited cell apoptosis *in vitro*

**Figure 3:**
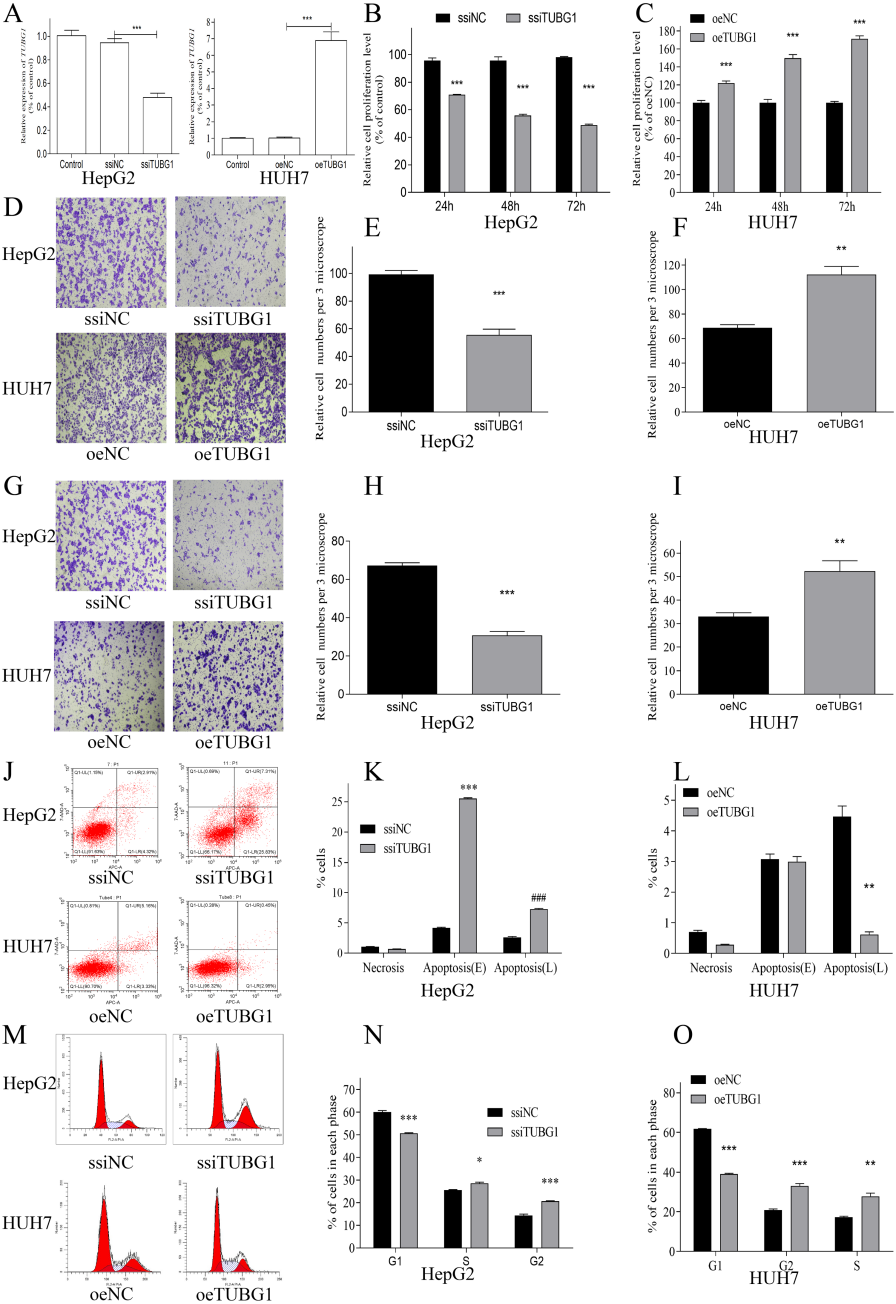
TUBG1 promoted cell proliferation, migration, and invasion, but inhibit cell apoptosis in HCC cells. (A) After TUBG1 knockdown/overexpressed, the mRNA expression level of TUBG1 has changed, in HepG2 and HUH7 cell lines, *** *P* < 0.001. (B, C) TUBG1 knockdown significantly inhibited the proliferation of HepG2 cells at different time periods, while TUBG1 overexpression promoted the proliferation of HUH7 cells, *** *P* < 0.001. (D–F) TUBG1 knockdown inhibited the migration and invasion (G–I) ability of HepG2 cells at 48 h, *** *P* < 0.001, while TUBG1 overexpression promoted the migration and invasion (G-I) ability of HUH7 cells at 48 h, *** *P* < 0.001. (J–L) TUBG1 knockdown promotes apoptosis in HepG2 cells, while TUBG1 overexpression inhibits apoptosis in HUH7 cells, ***### *P* < 0.001, and (M–O) promoted the G1/S checkpoint transition, * *P* < 0.05, *** *P* < 0.001. TUBG1, tubulin gamma 1; HCC, hepatocellular carcinoma; ssiNC, smart silence negative control; ssiTUBG1, smart silence TUBG1; oeNC, overexpressed negative control; oeTUBG1, overexpressed TUBG1.

**Figure 4:**
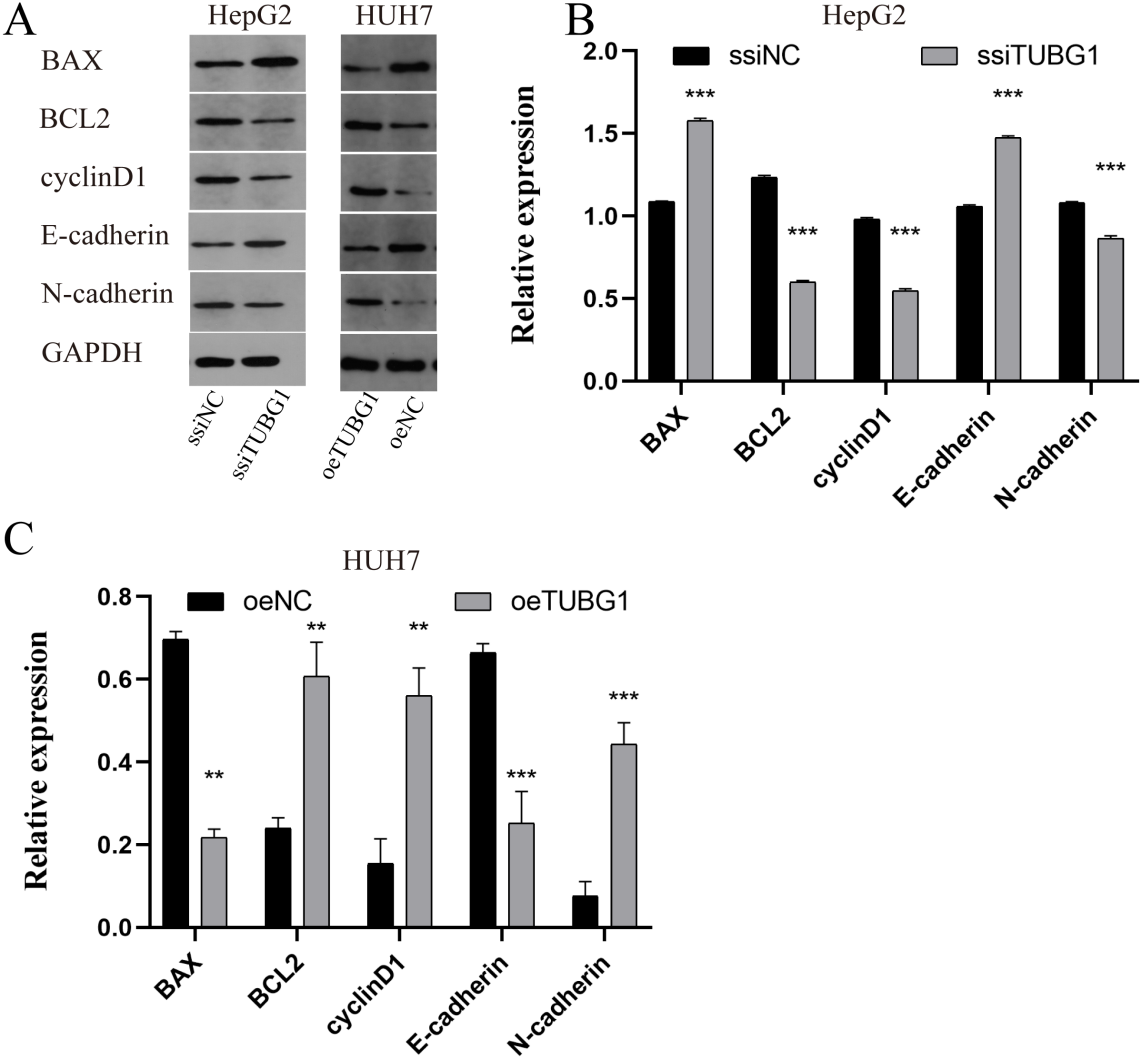
TUBG1 overexpression caused upregulation in the expression of BCL-2, cyclinD1, and N-cadherin and downregulation in the expression of Bax and E-cadherin. Also, TUBG1 knockdown has an adverse effect. ssiNC, smart silence negative control; ssiTUBG1, smart silence TUBG1; oeNC, overexpressed negative control; oeTUBG1, overexpressed TUBG1; *** *P* < 0.001.

In order to further verify the effect of TUBG1 on the malignant progression of HCC cell lines, HepG2 cells were selected for TUBG1 knockdown, and HUH7 cells were selected for TUBG1 overexpression (Fig. 3A) to detect cell proliferation, migration, invasion, and apoptosis, respectively. The results show that TUBG1 can effectively promote cell proliferation (Figs. 3B, 3C), migration (Figs. 3D–3F), invasion (Figs. 3G–3I) and inhibit cell apoptosis (Figs. 3J–3L) of HCC cells. And TUBG1 also promoted G1/S checkpoint transition (Figs. 3M–3O). TUBG1 overexpression caused upregulation in the expression of BCL-2, cyclinD1, and N-cadherin and downregulation in the expression of Bax and E-cadherin. And also, TUBG1 knockdown has an adverse effect (Figs. 4A–4C).

**Table 1:** Correlation between TUBG1 expression and clinicopathological characteristics in HCC (*n* = 371) TUBG1, tubulin gamma 1; HCC, hepatocellular carcinoma.

Characteristics		Total	TUBG1		*P*
			High	Low	
Age	≥50	300	148	152	0.998
	<50	70	37	33	
	NA	1	1		
Gender	male	251	126	125	0.530
	female	120	60	60	
Stage	I	170	65	105	<0.001
	II	87	51	36	
	III+IV	90	55	35	
	NA	24	15	9	
T	T1+T2	275	127	148	0.004
	T3+T4	94	59	35	
	NA	2		2	
Race category	Asian	158	89	69	<0.001
	white	184	85	99	
	other	19	8	11	
	NA	10	4	6	
Vital status	alive	240	108	132	0.006
	death	130	77	53	
	NA	1	1	0	

### TUBG1 probably involved in some pathways related to HCC occurrence and development

**Figure 5:**
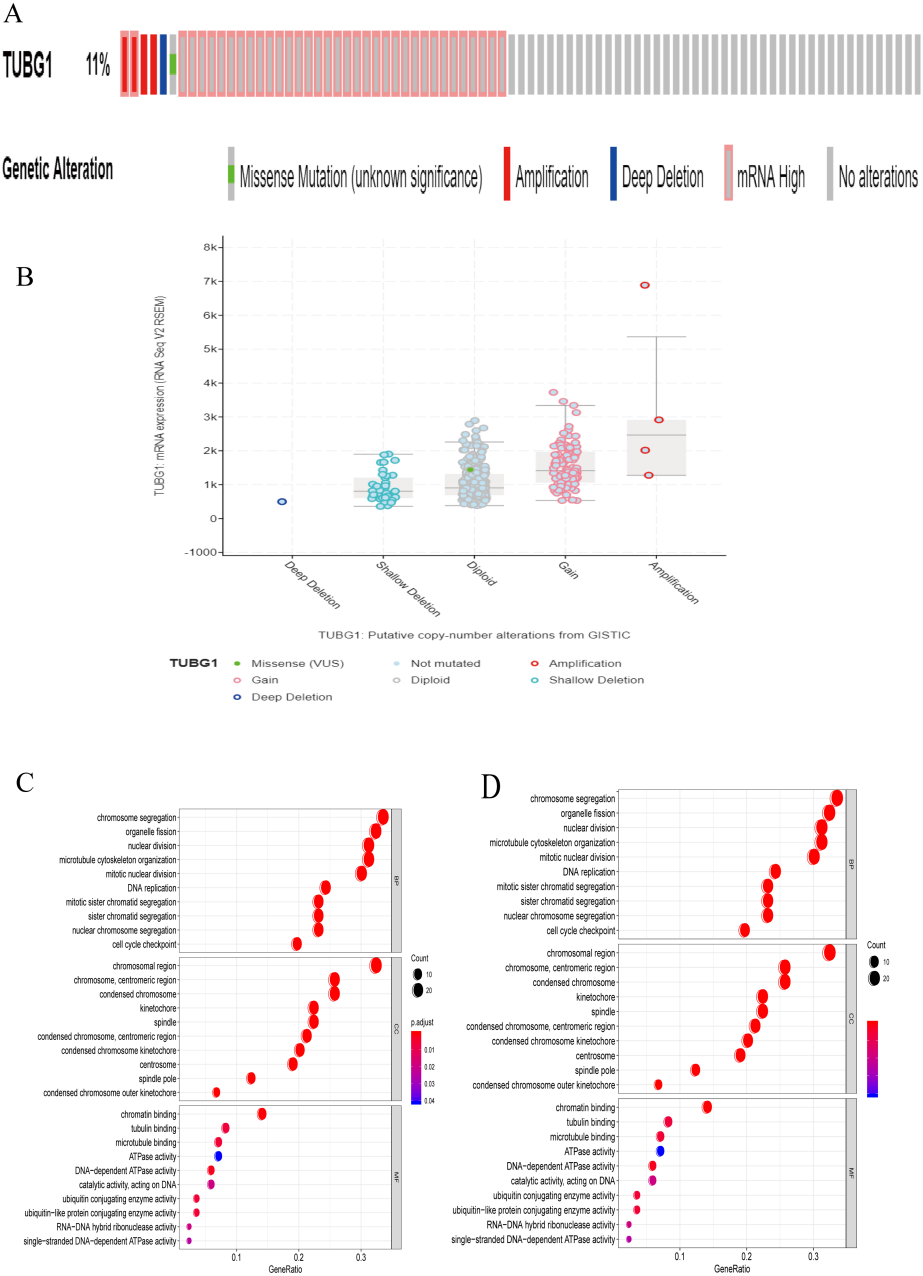
Analysis of the biological function of TUBG1. (A) Probability of different mutations in TUBG1; data is from the cBioprotal database. (B) Relationship between TUBG1 expression and different mutation types. (C) GO term analysis of co-regulated genes. *Y*-axis shows the GO terms of biological process. The length of the bars is proportional to the number of genes. (D) KEGG enrichment. The size of the nodes is proportional to the number of genes. TUBG1, tubulin gamma 1; HCC, hepatocellular carcinoma; GO, gene ontology; KEGG, Kyoto Encyclopedia of Genes and Genomes.

Using the cBioportal online database (https://www.cbioportal.org/) (Cerami et al., 2012; Gao et al., 2013), we found that 11% patients with HCC showed mutations in TUBG1 (Fig. 5A), and different types of mutations were associated with TUBG1 expression (Fig. 5B). There were 94 co-regulated genes (*r* > 0.6, *P* < 0.05) that were predicted to be target genes of TUBG1 (Table S1). To further explore the biological functions of TUBG1, we performed GO and KEGG analysis; GO term enrichment analysis revealed that co-regulated genes were enriched in biological processes mainly involved in chromosome segregation, chromosomal region, and chromatin binding (Fig. 5C). In addition, KEGG pathway analysis showed that co-regulated genes were mainly involved in cell cycle, oocyte meiosis, platinum drug resistance, and p53 signaling pathway (Fig. 5D). All these pathways were verified to be involved in multiple cell biological functions, including cell metabolism, apoptosis, and migration. These results suggested that TUBG1 plays some important roles in HCC cell proliferation and invasion, and it is even involved in conferring resistance to certain chemotherapy drugs.

## Discussion

HCC is difficult to diagnose in its early stages; only three widely used biomarkers exist at present: α-fetoprotein (AFP), core fucosylated AFP (AFP-L3), and des-gamma-carboxy prothrombin. All of them have shown some value in HCC detection, but with limited sensitivity (West, Black & Mehta, 2019). Therefore, there is an urgent need to identify sensitive and specific biomarkers for improving the diagnosis and for accurately evaluating the prognosis of HCC. Herein we report that TUBG1 plays a critical role in the occurrence and development of HCC and has a great value as a diagnostic and prognostic biomarker for HCC.

In the present study, we found that TUBG1 was overexpressed in HCC tissues as well as HCC cell lines. Similarly, earlier studies have reported high expression levels of TUBG1 in breast cancer (Watanabe et al., 2018), non-small cell lung cancer (Maounis et al., 2012), and medulloblastomas (Caracciolo et al., 2010). These findings indicate that although TUBG1 has a tendency of overexpression in a variety of tumors, it does not have strong tissue or organ specificity; thus, TUBG1 might be a universal oncogene. Furthermore, we found that the expression level of TUBG1 was closely related to clinicopathological characteristics (clinical stage, race, and survival status of the tumor), particularly to clinical stage and survival status, which are closely monitored in the clinical diagnosis and treatment process.

It appears that TUBG1 is a reliable diagnostic and prognostic biomarker for HCC. According to TCGA, we found that the AUC of TUBG1 was >0.9, with the value being superior to that of AFP (sensitivity of 41%–65% and specificity of 80%–94% for elevated serum AFP levels of >20 ng/mL in diagnosing HCC (Wong, Ahmed & Gish, 2015), which is one of the most common biomarkers for HCC diagnosis. In addition, the use of γ-tubulin to predict BRCA status showed sensitivity of 83% and specificity of 89% (Watanabe et al., 2018), suggesting that TUBG1 is a promising diagnostic biomarker for HCC and has potential value for diagnosing other cancers. Moreover, we found that upregulated expression levels of TUBG1 were associated with shorter OS and DFS in patients with HCC, implying that TUBG1 can serve as a biomarker for predicting HCC progression. SOX9 positivity was associated with shorter DFS according to a study by Kawai et al. (2016) who reported that SOX9 is a novel HCC/cancer stem cell marker regulating the Wnt/beta-catenin pathway and its downstream target, osteopontin; patients with SOX9^+^ tumors exhibited significantly poorer recurrence-free survival, but no significant difference was observed in OS. TUBG1 probably a more accurate predictor of prognosis in HCC patients. In addition, Dementyeva et al. (2013) found that in case of multiple myeloma, patients with positive centrosome amplification showed lower TUBG1 expression levels, reporting better prognosis for centrosome amplification-positive newly diagnosed patients. This implies that TUBG1 can predict prognosis for conditions other than HCC.

TUBG1 is involved in HCC occurrence and development. TUBG1 promoted cell proliferation, migration, and invasion and inhibited cell apoptosis *in vitro*. At the same time, its expression was related to the clinical stage, race, and survival status of the tumor, suggesting that high expression levels of TUBG1 may lead to poor prognoses. These results suggest that TUBG1 plays a key role in the progression of HCC. GO term enrichment analysis revealed that co-regulated genes were enriched in biological processes mainly involved in chromosome segregation, chromosomal region, and chromatin binding. Moreover, KEGG pathway analysis showed that co-regulated genes were mainly involved in cell cycle, oocyte meiosis, platinum drug resistance and p53 signaling pathway. These pathways have been commonly associated with tumorigenesis (Vogelstein, Lane & Levine, 2000; Zarrinpar, 2017). This also explains to a certain extent why TUBG1 expression was related to the proliferation, invasion, migration, cell cycle, and apoptosis of HCC cells. To support us *in vitro* results, pertinent *in vivo* experiments need to be performed in future studies.

We suggest that aberrant expression of TUBG1 probably responsible for the aforementioned effects for the following reasons. A reasonable explanation could be that γ-tubulin is involved not only in microtubule nucleation and organization but also, surprisingly, in the coordination of prometaphase events such as chromosome segregation and septation during cytokinesis (Hendrickson et al., 2001). In some *in vivo* studies, it was observed that TUBG1 knockout mice died at the blastocyst stage (Vinopal et al., 2012); the most likely explanation for this is that the development of TUBG1-deficient embryos stopped at the morula/blastocyst stages due to a characteristic mitotic arrest. This finding supports a series of cell cycle and apoptosis changes caused by TUBG1 knockdown to a certain extent (Yuba-Kubo et al., 2005). Further, the above evidence indicates that TUBG1 has important biological functions when it is optimally expressed. However, we found a certain degree of variation in the expression of TUBG1 in patients with HCC. The most common types were gain and diploid, and the expression abundance of TUBG1 was closely related to tumor prognosis. Therefore, we believe that aberrant expression of TUBG1 leads to poor prognosis of HCC. Nevertheless, further studies as warranted so as to identify a clear molecular biological mechanism underlying TUBG1 carcinogenesis.

## Conclusions

We report that TUBG1 is an important oncogene in HCC. It promotes HCC progression and may serve as a potential prognostic biomarker for HCC. Future studies are warranted to unveil molecular biological mechanisms underlying TUBG1 carcinogenesis.

##  Supplemental Information

10.7717/peerj.14415/supp-1 Supplemental Information 1 The original exposures of the genes co-expressed with the TUBG1 gene and the western blot generated in this study

10.7717/peerj.14415/supp-2 Supplemental Information 2 TUBG1 expression levels in various cell lines The images are from the Human Protein Atlas database CC BY 3.0. The original file is available at: https://www.proteinatlas.org/ENSG00000131462-TUBG1/cell.

## Additional Information and Declarations

### Data Availability

The following information was supplied regarding data availability: The original Western blots are available in the Supplemental Files. The raw data is available in Figshare: Zhang, Kainan (2022): fig3RAW.zip. figshare. Figure. https://doi.org/10.6084/m9.figshare.20346897.v1.
