# Supplementary figures and images for: Upregulated TUBG1 expression is correlated with poor prognosis in hepatocellular carcinoma
Source: PeerJ. 2022 Dec 5;10:e14415. doi: 10.7717/peerj.14415 (PMC9745943; doi:10.7717/peerj.14415)

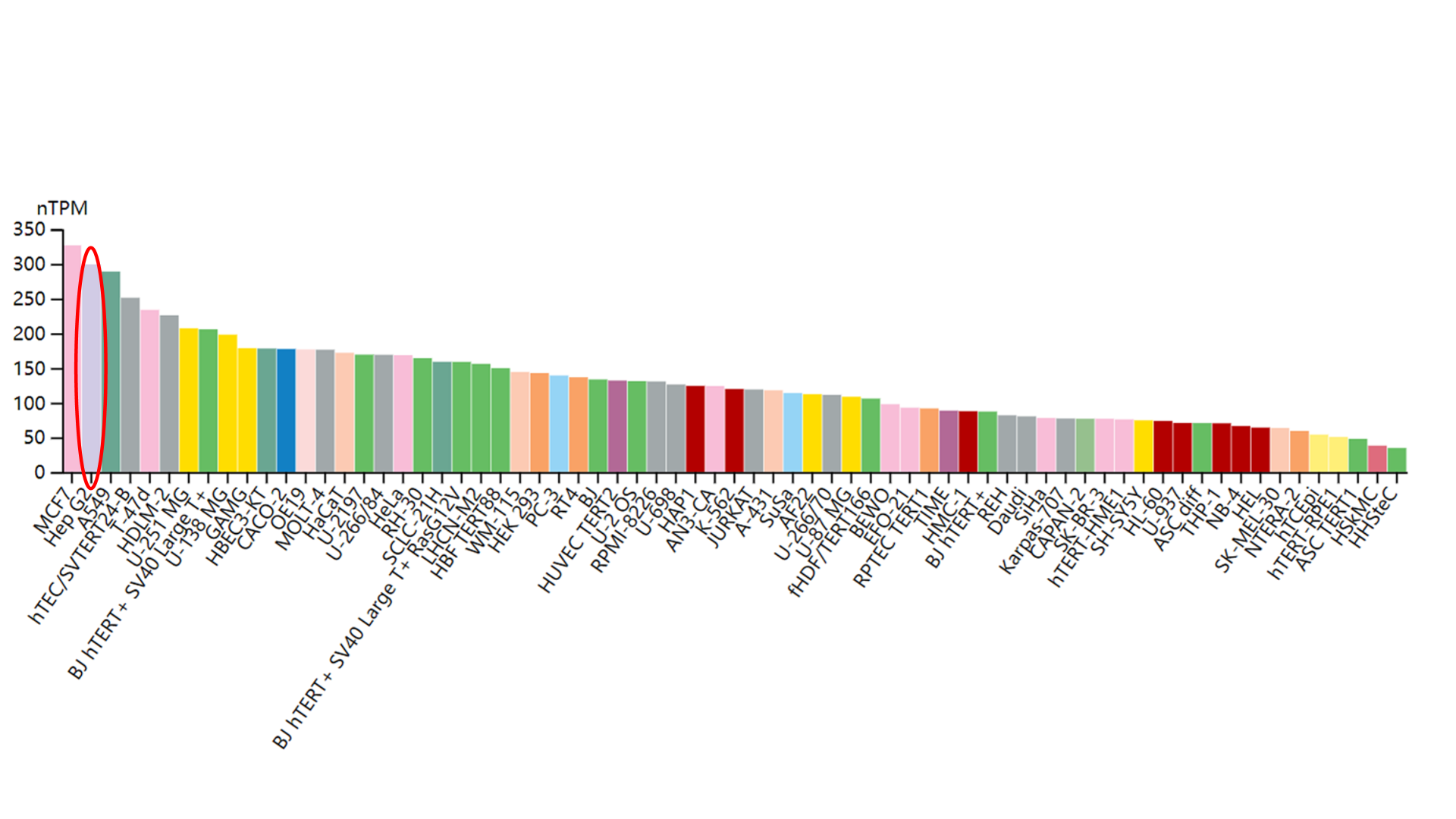

Supplement: Supplemental Information 2 — The images are from the Human Protein Atlas database CC BY 3.0. The original file is available at: https://www.proteinatlas.org/ENSG00000131462-TUBG1/cell. [file peerj-10-14415-s002.png]
